# Anticipatory effect of execution on observation: an approach using ExoPinch finger robot

**DOI:** 10.3906/sag-1812-143

**Published:** 2019-08-08

**Authors:** Kutluk Bilge ARIKAN, Hassan Gol Mohammad ZADEH, Ali Emre TURGUT, Murat ZİNNUROĞLU, Gözde BAYER, Zafer GÜNENDİ, Bülent CENGİZ

**Affiliations:** 1 Department of Mechanical Engineering, Faculty of Engineering, TED University, Ankara Turkey; 2 Department of Mechatronics Engineering, Faculty of Engineering, Atılım University, Ankara Turkey; 3 Department of Mechanical Engineering, Faculty of Engineering, Middle East Technical University, Ankara Turkey; 4 Department of Physical Medicine and Rehabilitation, Faculty of Medicine, Gazi University, Ankara Turkey; 5 Department of Neurology, Faculty of Medicine, Gazi University, Ankara Turkey

**Keywords:** Mirror neurons, action observation, anticipation

## Abstract

**Background/aim:**

This study aims to explore the mirror neuron system (MNS) involvement using mu (8–12 Hz)/beta (15–25 Hz) band suppression in an action observation-execution paradigm.

**Materials and methods:**

Electrophysiological (EEG) data from 16 electrodes were recorded while 8 participants observed video clips of a hand squeezing a spring. Specifically, the effect of anticipated execution on observation was studied. For this purpose, a fully actuated finger exoskeleton robot was utilized to synchronize observation and execution and to control the execution condition for the participants. Anticipatory effect was created with a randomized robot accompany session.

**Results:**

The results showed that the observational condition (with or without anticipation) interacted with hemisphere at central channels near somatosensory cortex. Additionally, we explored the response of MNS on the kinetics features of visual stimuli (hard or soft spring).

**Conclusion:**

The results showed an interaction effect of kinetics features and hemisphere at frontal channels corresponding nearly to the ventral premotor cortex area of the brain. The activation of mirror neurons in this area plays a crucial role in observational learning. Based on our results, we propose that specific type of visual stimuli can be combined with the functional abilities of the MNS in the action observation based treatment of hand motor dysfunction of stroke patients to have a positive additional impact.

## 1. Introduction

An impaired hand function is often reported to be the most disabling motor deficit after stroke. The functional reuse of the hand is of paramount importance for the patient’s physical independence and social integration. Therefore, the focus of stroke rehabilitation has been mainly the treatment of the proximal and distal segments of the upper limb, especially the hand motor dysfunction [1,2].

Different rehabilitation approaches for this neurological handicap have been proposed; among them action observation (AO) treatment could be a viable strategy to improve motor rehabilitation following stroke [3–5]. During a typical session in AO treatment, patients observe a meaningful action performed by an agent and afterwards they perform the observed motor act at the best of their ability [3]. AO-based rehabilitation approach has the potential that the treatment can be tailored to a specific need of a single patient. For example, the type and amount of visually presented actions (stimulations) can be well defined according to the patients’ needs.

It is now a well-accepted notion that in human brain, motor areas are recruited not only when actions are executed but also when they are observed [1]. The neurophysiological basis for this execution-observation matching system relies on the discovery of the mirror neuron system (MNS) [6,7]. The observed or seen action seems to be reflected by neurons with mirror properties in the motor representation for the same action of the observer. This neural network supporting the action observation and execution processing corresponds to a set of areas in parietal, frontal and sensorimotor cortices [8] and has been proposed to subserve as a key neural basis for action understanding [9–11], social communication and motor learning [12].

Observation of action exploits this neural mechanism by activating the physiological network of motor areas where motor representations of observed actions are known to be present. In a study, observation of another person learning a novel reaching task affected the subsequent performance of naive observers [13]. Moreover, observation of a simple intransitive movement of the index and middle fingers increased the excitability of the motor cortex [14]. These and several other studies demonstrate that AO produces an increase in the excitability of the corticospinal pathways [15,16].

The applied therapeutic strategies in AO treatment may have a crucial role to recruit more deeply the related motor representations. Several of these approaches utilize the capacity of MNS to enhance recovery of the upper limb after stroke and assume that MNS actively participate in the process. Along this line, it was demonstrated that showing video clips of daily actions and subsequent imitation of these actions resulted in a significant improvement of motor functions in the experimental group of patients [17]. Indeed, when the observed action belongs to the motor repertoire of the observer, the putative MNS can match the observed action on the neural structures involved in its execution and can activate previously learned movements [18,19]. Furthermore, the observation of hand-object interactions allows the observer to code the intentions of individuals performing the observed actions [6]. The same mirror neuron mechanism serves the decoding of the immediate scope of that action. Understanding the intention beside the observed action is an important component that allows for the preparation of the action execution system [20,21]. Overall, these findings suggest the value of showing actions that are common to the observer in context to ameliorate the therapeutic effects in AO treatment. 

Thus far, AO has been combined with subsequent physical practice as a new neurorehabilitative program. The observation of actions with concomitant physical training of the observed actions provides a significant improvement of motor functions after stroke [4,22]. Additionally, recent evidence suggest that when action observation and training exercises are performed simultaneously, the effects of action observation may increase, as it is known that corticomotor excitability is stronger when the observed action kinematically (spatially and temporally) matches the direction of the physical training [22–24]. The corticospinal excitability is also modulated when anticipating to see a movement and there will be a preparation for a possible motor response prior to the onset of the movement subsequently observed [25]. Since the observation of movement and execution of that movement share common neural processes [26,27], anticipatory changes in motor cortical neural activity also occur for an expected movement [28,29]. These findings demonstrate that expectancy of an upcoming action automatically activates the motor system. 

Among the methods used for inferring human MNS activity are power changes in the electroencephalogram (EEG) alpha (8–12 Hz) and beta (15–25 Hz) bands primarily over the sensorimotor areas [1, 2,30]. In EEG, rhythms in the alpha and beta frequency ranges are typically accompanied by a power decrease (reflecting cortical activity) in response to both execution and observation of an action [31–34]. This decrease in alpha band, in reference to a baseline condition is known as mu rhythm desynchronization [35–37].

Our study model focuses on the core and the extended areas of the putative MNS at both mu and beta frequency bands. For this purpose, human EEG cortical oscillatory activities were measured at electrode locations that nearly correspond to the areas of this system [8]: somatosensory and ventral premotor cortex plus the inferior parietal lobule. 

The major novelty of this study was to assess whether neural activity during action observation can be enhanced by an anticipatory effect of execution when this actual movement is externally generated by a robotic system. The motivation behind the present study was to determine the degree to which the efficiency of action observation might achieve for treatment of stroke. For this purpose, given the significance of robot-induced technology for poststroke rehabilitation [2], a finger exoskeleton robot was utilized to fully synchronize the action observation with execution in an experimental session. The random robot accompany (i.e., execution of the observed action) to observation in the subsequent session allowed us to create a unique scenario in which the execution becomes an anticipatory concurrent factor of the observation. In the current experiment, it was hypothesized that the effect of anticipation would change the functional activation in the areas of the MNS.

This methodology makes use of a robot-guided execution of an action rather than a self-initiated action and the results of this study will have an additional positive effect over an AO-based rehabilitation protocol. 

## 2. Materials and methods

### 2.1. Subjects

A total of 8 right-handed volunteers (all males, mean age = 35.8, SD = 9.3) without a neurological illness or a history of upper limb injury participated in this study. The participants had normal or corrected-to-normal vision. Subjects were informed about the procedure before the experiment and informed consent was obtained from each participant in the study. The experimental procedure was approved by the local Ethics Committee.

### 2.2. Stimuli and procedure

**Figure 1:**
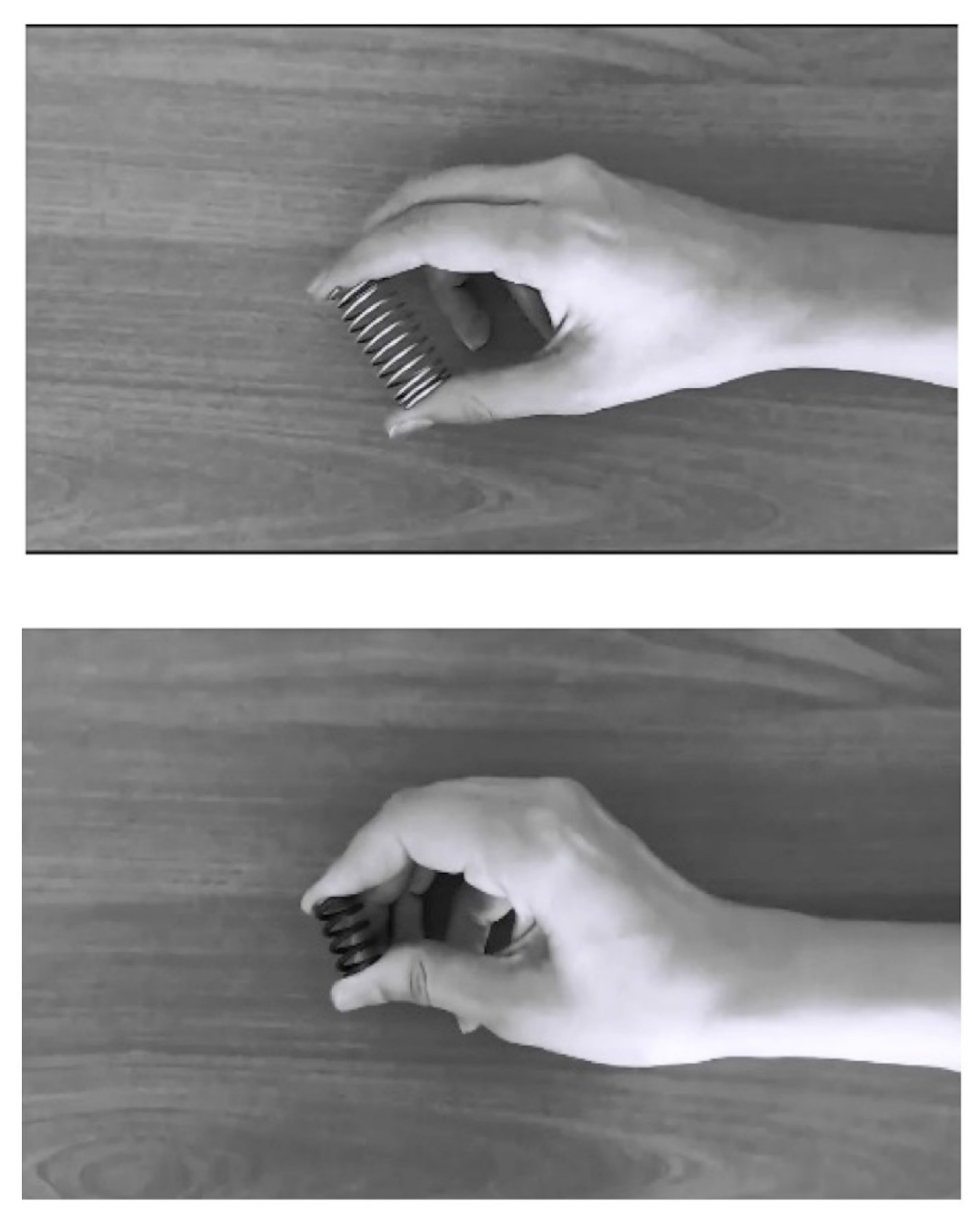
Still frames from the videos used in the experiment depicting the squeezing the hard spring (hs, bottom) and soft spring (ss, up).

Subjects sat on a comfortable chair 1 m away from a computer screen (18” LCD monitor at 60 Hz). Stimuli were video clips of actions performed by the left hand of the experimenter. Video recordings were converted to gray-scale and clipped such that the motion of the hand began at the first frame of each video. A video clip lasted for 2 s and one session of the experiment consisted of 40 trials. The stimuli were videotaped with the same background and hand/wrist position on the screen (Figure 1). A black screen of 4 s was presented between each video as a baseline condition. A short break was provided to the subjects between the sessions.

Before starting EEG recordings, participants were presented with the objects used in the video stimuli. EEG data were recorded during 3 successive sessions: in the first session (obs), subjects merely observed videos of the hand squeezing and releasing a hard or a soft spring (Figure 1). These conditions will be referred to as hs and ss, respectively.

**Figure 2:**
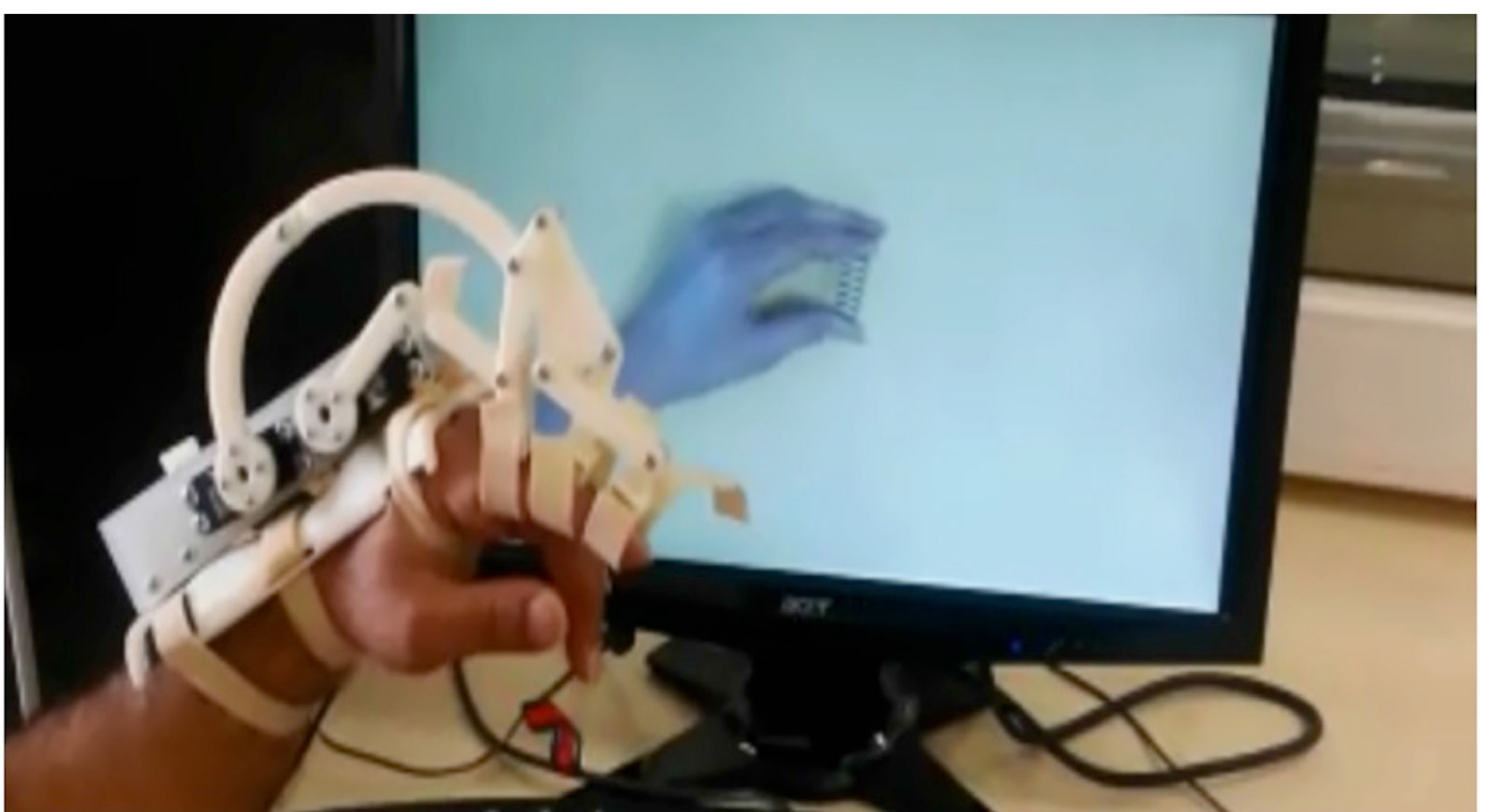
A still frame from session 2 (obs+exe condition): simultaneous action observation and action execution with exoskeleton robot ExoPinch.

In the second session (obs+exe), the fully actuated (with 2-degrees of freedom) exoskeleton finger robot ExoPinch simultaneously accompanied the left hand’s index finger of the subjects with each observed movement presented in the video clip (Figure 2). 

The joints of the robotic system are aligned with the joints of the human index finger such that the enclosed index finger is manipulated by the robot. For the precise synchronization of the robot movement with the observed one, 5 main steps were followed prior to the experiment: 1. each type of video clip (hs, ss) was decomposed into their video frames (Asus 2 GB NVIDIA GTX 128 bit Graphics card) by PsychoPy2 software [38]. 2. Several frames were selected including the initial position, final position, and some intermediate positions in between. Care was taken to ensure that the chosen frames were able to basically define the 2 s movement. 3. Time elapsed for the selected frames were obtained by PsychopPy2 software [38]. 4. Kinematic motion analysis of the selected frames was performed by Tema Trackeye 3.5 motion analysis software (Image Systems AB). The angles were measured from the distal and proximal interphalangeal joints of the index finger during squeezing of the spring. 5. Both the temporal and angular data (obtained from steps 3 and 4) of the selected frames were adapted to Matlab Simulink (The MathWorks, Inc., Natick, MA, USA) environment for the precise synchronization of the robot finger ExoPinch with the visual stimuli. 

In session 3, ExoPinch accompanied the observed movements but in a randomized paradigm created by a separate code using PsyhcoPy2 software. Neither the subject nor the experimenter knew which trials were mere observations (without robot accompany) or combination of robot execution with observation. These experimental conditions will be referred to as *no robot *(robot did not accompany) and* robot *(robot accompanied), respectively. The experimental conditions were detailed in Table. 

**Table:** Sessions of the study

Session	Condition	Observation	Execution	Anticipation	Simultaneous
1	obs	✔			
2	obs+exe	✔	✔		✔
3	robot	✔	✔	✔	✔
3	no robot	✔		✔	

In session 2 and 3, subjects were told to keep their hand relaxed in order not to interfere with ExoPinch’s regular motion execution. In all sessions, the experimental conditions were chosen to be strictly associated with the observation (with or without execution) of hand-object interaction, given the high responsiveness of the MNS to object directed hand actions. The visual appearance of stimuli and continuous EEG recording were synchronized with PsychoPy2 software. Visual stimuli in each session were presented in a randomized order ensuring that each participant experienced a different randomized sequence of trials. 

### 2.3. Robot control, electrophysiological (EEG) recording and data analysis

**Figure 3:**
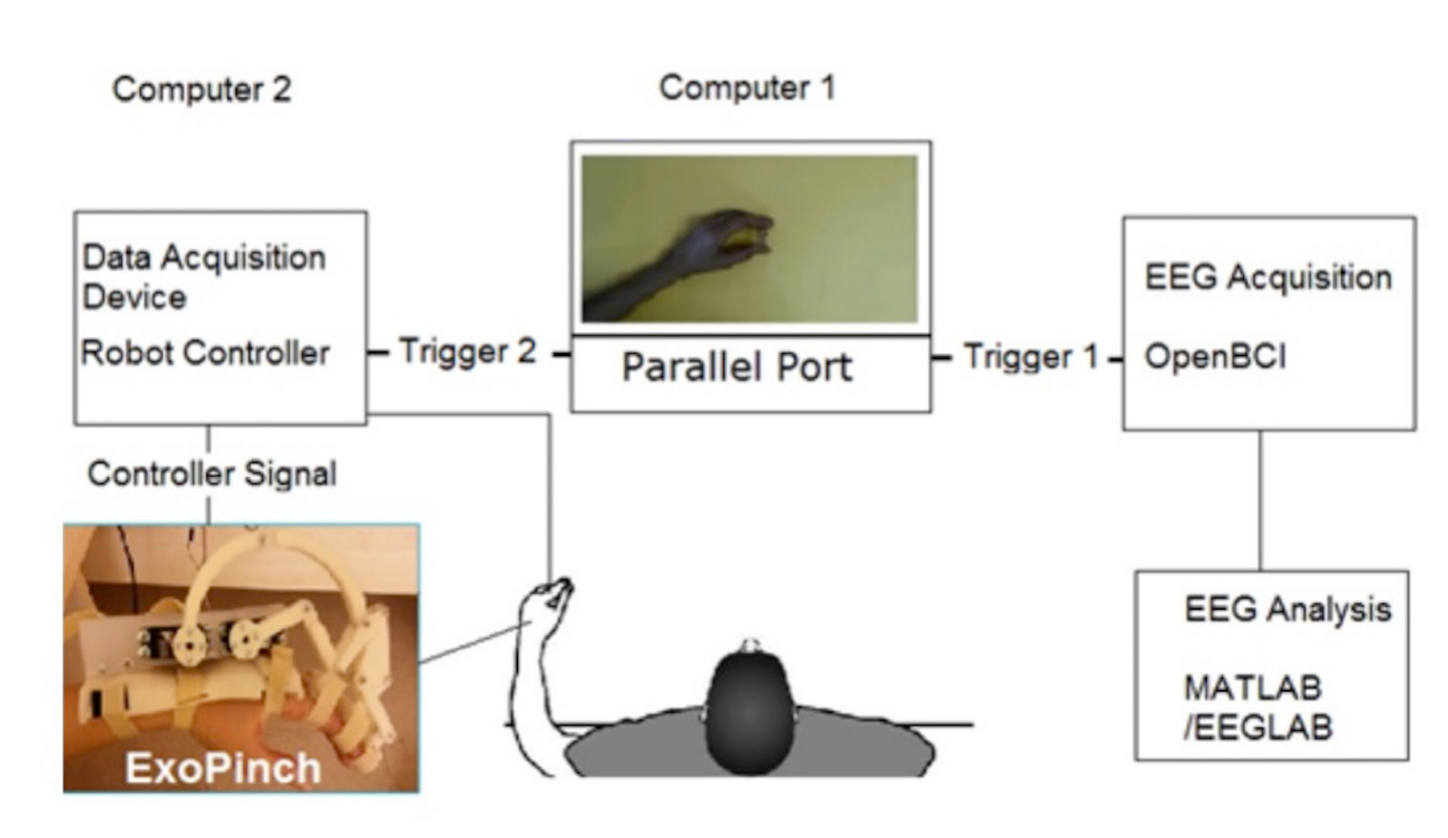
Graphic representation of the experimental set up. Computer 1 presents the visual stimuli and records the EEG data; computer 2 controls the robot movement.

The experimental setup utilized two separate computers (3.2 GHz CPU, 8 GB RAM), one for the visual stimuli and EEG acquisition and the other for the finger robot control (Figure 3).

Computer 1 in Figure 3 presented the visual stimuli and recorded the EEG data stream. On the other hand, it served as a trigger signal station; onset of the each visual stimulus was marked in EEG acquisition device by computer 1 via its parallel port utility (Trigger 1 in Figure 3). Computer 2 initiated the robot finger upon receiving the signal from the parallel port of Computer 1 (Trigger 2 in Figure 3). It has to be emphasized that separate data pins for the trigger signals of the parallel port were enabled to discriminate the task for the robot. The termination of ExoPinch accompany was accomplished by the previously defined (kinematically) movement pattern in Computer 2.

The electroencephalogram (EEG) was recorded continuously (bandpass, 0.1–100 Hz; sampling rate, 250 Hz) with the 32 bit board OpenBCI including the Daisy module with a 16 channel system. UltraCortex Mark 4 dry electrode headset was used conforming international 10–20 electrode placement: Fp1, Fp2, C3, C4, F3, F4, F7, F8, T3, T4, T5, T6, P3, P4, O1, and O2. Electrode contact resistances were confirmed to be less than 5 kΩ using real time electrode impedance measurements provided by an open project Processing 2.2.1. A Matlab (The MathWorks, Inc., Natick, MA, USA) code is generated to process and sort out the raw EEG data. EEG data were then preprocessed using EEGLAB 13.4.4b MATLAB (The MathWorks, Inc., Natick, MA, USA) toolbox [39].

A linear finite impulse response (FIR) filter from 1 Hz to 40 Hz was applied to eliminate the baseline drifts and the 50 Hz line noise. A common average reference (CAR) was performed on all 16 electrodes in order to produce the reference free EEG data. The CAR method provides a better signal-to-noise ratio for the mu or beta-rhythm than does any standard (e.g. ear-reference) method [31]. Data were epoched ranging from 1000 ms preceding stimulus onset (appearance of hand object interaction on the screen) to 2000 ms after stimulus onset and were time locked to the onset. In each epoch, baseline was considered as the period starting 1000 ms before the onset of stimulus and ending at the stimulus onset. Atypical epochs were removed from further analysis by applying improbability test with standard deviation ≥6 to remove eye blink, electrical change, and muscle movement. After preprocessing, averaged data (n = 8 subjects) were analyzed for each condition in the time frequency domain at all 16 channels. EEG spectra were decomposed using a 3-cycle wavelet with the baseline corrected event related spectral perturbation (ERSP) method [38]. The mean power of the baseline period was removed from the power at each time point of the experimental trials. Both the spectral and the time windows of mu/beta oscillations for statistical analysis were determined from ERSP data plotted in EEGLAB/MATLAB environment. The mean mu/beta band power values (in dB) were extracted at a number of frontal (F7, F8), central (C3, C4) and parietal (P3, P4) channels since these regions almost exclusively included regions that have been associated with the MNS in the literature: d/vPMC, Brodmann area 44/2 and IPL [34, 40,41]. Each channel with the experimental condition was carefully analyzed in ERSP time-frequency plots for the specific time window of desynchronization of mu/beta band power before entering the data into statistical analysis. 

Significance of mu/beta band power desynchronization against zero (baseline) for all conditions was analyzed using t-test with P value set at 0.05. P values reported below are two-tailed except for the comparisons of mu and beta power against zero, where results are one-tailed since a decrease in mu/beta power was expected. The Shapiro–Wilk test was applied to assess whether data were likely from a normal distribution. Although data were obtained from 16 electrodes across the scalp, the focus has been on the relevant channels (C3, C4, F7, F8, P3 and P4) and significance of mu/beta desynchronization was reported over the somatosensory cortex (C3 and C4), given the prior literature.

In statistical analysis of any anticipatory effect, our main comparison of interest was based on two main assumptions: condition obs in session 1 was matched to no robot condition in session 3 and similarly, condition obs + exe in session 2 was matched to robot condition in session 3. Indeed, both in obs and no robot conditions, subjects merely observed the video stimuli, but they knew that there was a possibility of robot accompany in no robot condition. Similarly, both conditions, obs + exe in session 2 and robot condition in session 3, were observation of action with execution. This methodology with our experimental paradigm allowed us to analyze whether a possible anticipation of execution during observation of an action (no robot condition in session 3) facilitates the mirror neuron activity more effectively than the mere observation of that action without any anticipation (obs condition in session 1). It is also possible that neural oscillations (mu/beta suppression) during action observation with execution (obs + exe condition in session 2) might be modulated when execution was unexpectedly present (robot condition in session 3). Therefore, two separate (2 (condition) × 2 (hemisphere)) repeated measures ANOVA were performed for conditions (obs, no robot; obs+exe, no robot) and hemisphere (right, left) over central (C3, C4), frontal (F7, F8) and parietal channels (P3, P4). Mauchly’s test indicated that the assumption of sphericity was not violated (P > 0.05).

Although our primary interest was to analyze the anticipatory effect on observation, additionally, EEG oscillatory activity in mu/beta frequency band was analyzed from the ERSP plots for the hand movement with different kinetic conditions (hs and ss) in session 1, on the frontal areas to explore any modulation that may be specific to kinetic features of the action [42–45]. Before starting EEG recordings, subjects were presented with the objects used in the video clips and were asked to practice their stiffness (hard or soft). The respond of MNS is stronger to observed actions for which the subject has familiarity or he/she has a prior practice with the visual stimuli (46). It is therefore plausible to think that the mu suppression is a measure of the resonance between observed kinetics and those of the observer. Taken together, the observed actions were in their personal motor repertoire [19].

For the first session (obs) of the experiment, ERSP data (in dB) were extracted for two separate observational conditions (hs, ss) at the group level (8 participants). However, for completeness, the results from the parietal and central channels were also reported to cover the putative MNS on the scalp. Otherwise, conditions hs and ss in session 1 were combined into mere observation condition (obs) for our main discussion on the anticipatory effect.

## 3. Results

### 3.1. Comparison of obs and obs + exe conditions

**Figure 4:**
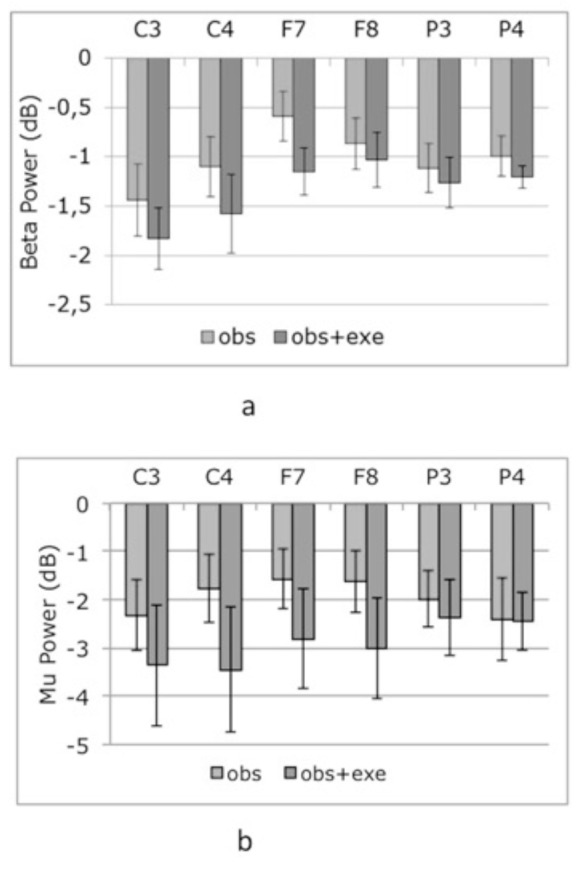
Mu (a) and beta (b) band suppression to experimental conditions. Bars represent the mean log ratio of power in the mu (8-12 Hz) and beta (15-25 Hz) frequency bands during session 1 (obs) and session 2 (obs+exe). Error bars indicate the standard error of the mean. A log ratio of less than zero indicates mu/beta suppression.

Suppression of mu/beta band power was observed in central (C3, C4) frontal (F7, F8), and parietal (P3, P4) channels. All the relevant channels showed slightly larger desynchronization values (in dB) for obs + exe condition (Figure 4).

At channels C3 and C4, T-tests comparing mu/beta suppression during each of the experimental conditions (obs, obs+exe) to zero showed significant suppression from baseline in mu [Condition: obs; C3 t(7) =* –*3.21, P = 0.008; C4 t(7) = *–*2.54, P = 0.02. Condition: obs + exe; C3 t(7) = *–*2.68, P = 0.016; C4 t(7) = *–*2.64, P = 0.017] and beta band (Condition: obs; C3 t(7) = *–*3.96, P = 0.003; C4 t(7) = *–*3.63, P = 0.004 Condition: obs+exe; C3 t(7) = *–*5.92, P = 0.0005; C4 t(7) = *–*3.96, P = 0.003) oscillations (Figure 4 a,b).

### 3.2. Comparison of no robot and robot conditions 

**Figure 5:**
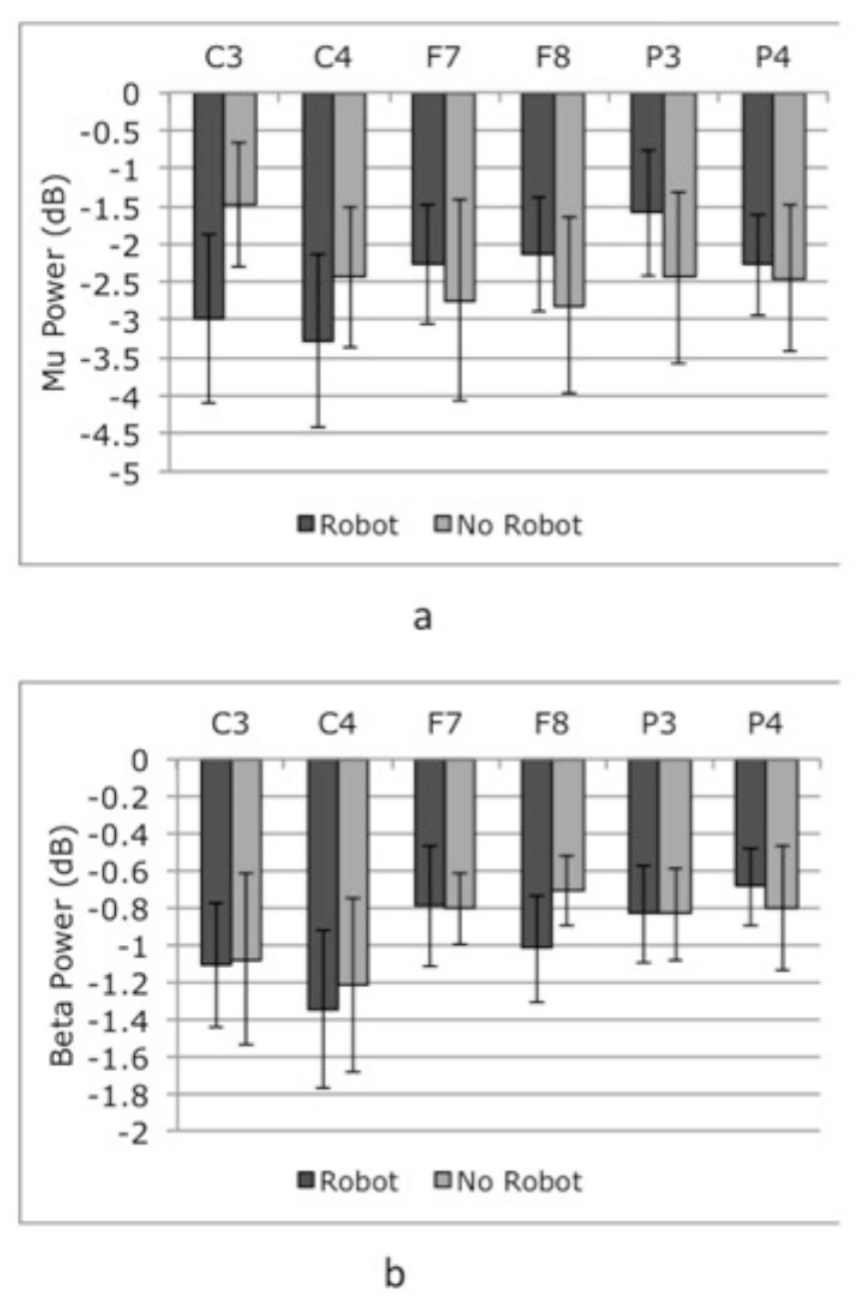
Mu (a) and beta (b) band suppression to experimental conditions. Bars represent the mean log ratio of power in the mu (8–12 Hz) and beta (15–25 Hz) frequency bands during session 3 (robot, no robot). Error bars indicate the standard error of the mean. A log ratio of less than zero indicates mu/beta suppression.

Suppression of mu/beta band power was observed in central (C3, C4) frontal (F7, F8), and parietal (P3, P4) channels with greater suppression of beta band at central channels (Figure 5). Desynchronization of mu/beta band (in dB) was slightly larger for robot condition over C4, contralateral to the movement (Figure 5).

At channels C3 and C4, T-tests comparing mu/beta suppression during each of the experimental conditions (no robot, robot) to zero showed significant suppression from baseline in mu (Condition: no robot; C3 t(7) = –1.83, P = 0.05; C4 t(7) = –2.61, P = 0.02. Condition: robot; C3 t(7) = –2.70, P = 0.016; C4 t(7) = –2.85, P = 0.013) (Figure 5a) and beta band (Condition: no robot; C3 t(7) = –2.35, P = 0.03; C4 t(7) = –2.61, P = 0.018 Condition: robot; C3 t(7)=-3.33, P = 0.007; C4 t(7)=-3.15, P = 0.008) oscillations (Figure 5b).

### 3.3. Effect of anticipation on observation and execution

Anticipation and observation

**Figure 6:**
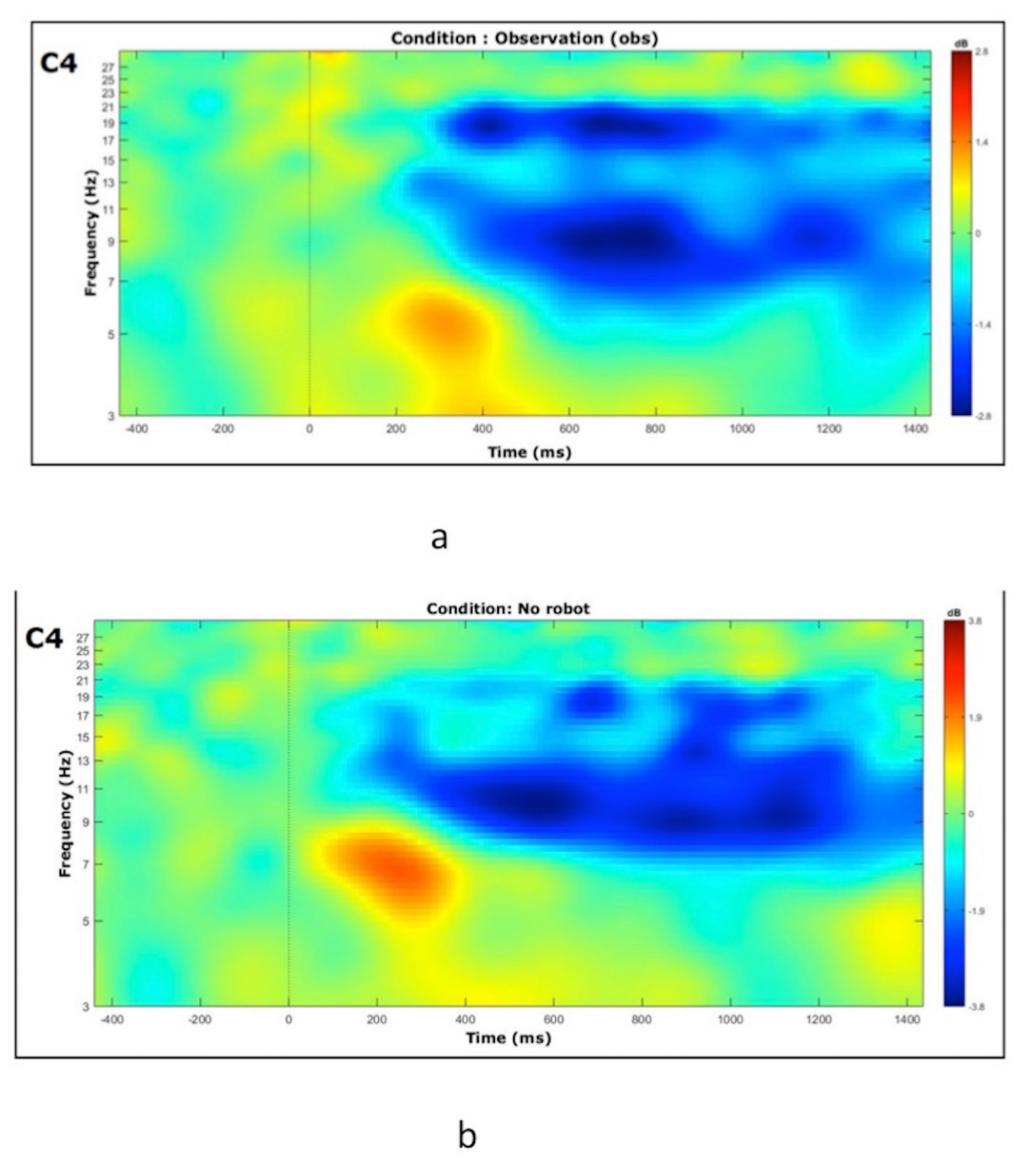
Time-frequency plots for two conditions (obs (a) and no robot (b)) at channel C4 (right hemisphere). Plots for the left hemisphere (C3) were similar and are not shown. The frequency axis is log scaled. The zero point on the time axis indicates the onset of the video stimuli.

Over the somatosensory cortex, time-frequency analysis of the relevant channels led to a decrease in mu (8–12 Hz) and beta (15–25 Hz) band power shortly after stimulus onset starting around 300 ms (Figure 6). 

In mu band, a 2 (observational condition: obs vs. no robot) by 2 (hemisphere: left vs. right) within subjects analysis of variance over central channels (C3, C4) revealed no significant main effects of observational condition [F(1,7) = 0.015, P > 0.05] nor hemisphere [F(1,7) = 0.367, P > 0.05], but there was a significant presence of observational condition by hemisphere interaction [F(1,7) = 8.161, P < 0.05]. Similar 2 (observational condition) by 2 (hemisphere) repeated measures ANOVAs at frontal (F7, F8) and parietal channels (P3, P4) revealed no main effects or interactions {F7–F8: observational condition [F(1,7) = 1.806], hemisphere [F(1,7) = 0.089], observational condition x hemisphere [F(1,7) = 0.001]; P3–P4: observational condition [F(1,7) = 0.102], hemisphere [F(1,7) = 0.725], observational condition x hemisphere [F(1,7) = 0.219], all P > 0.1}.

For beta frequency band, a similar 2 (observational condition: *obs* vs. *no robot*) by 2 (hemisphere: left vs. right) repeated measures ANOVAs were utilized. There were no main effects or interactions {C3–C4: observational condition [F(1,7) = 0.095], hemisphere [F(1,7) = 1.195], observational condition x hemisphere [F(1,7) = 3.048]; F7-F8: observational condition [F(1,7) = 0.015], hemisphere [F(1,7) = 0.515], observational condition x hemisphere [F(1,7) = 3.252]; P3–P4: observational condition [F(1,7) = 0.530], hemisphere [F(1,7) = 0.007], observational condition x hemisphere [F(1,7) = 0.483], all P > 0.1}.

Anticipation and execution

**Figure 7:**
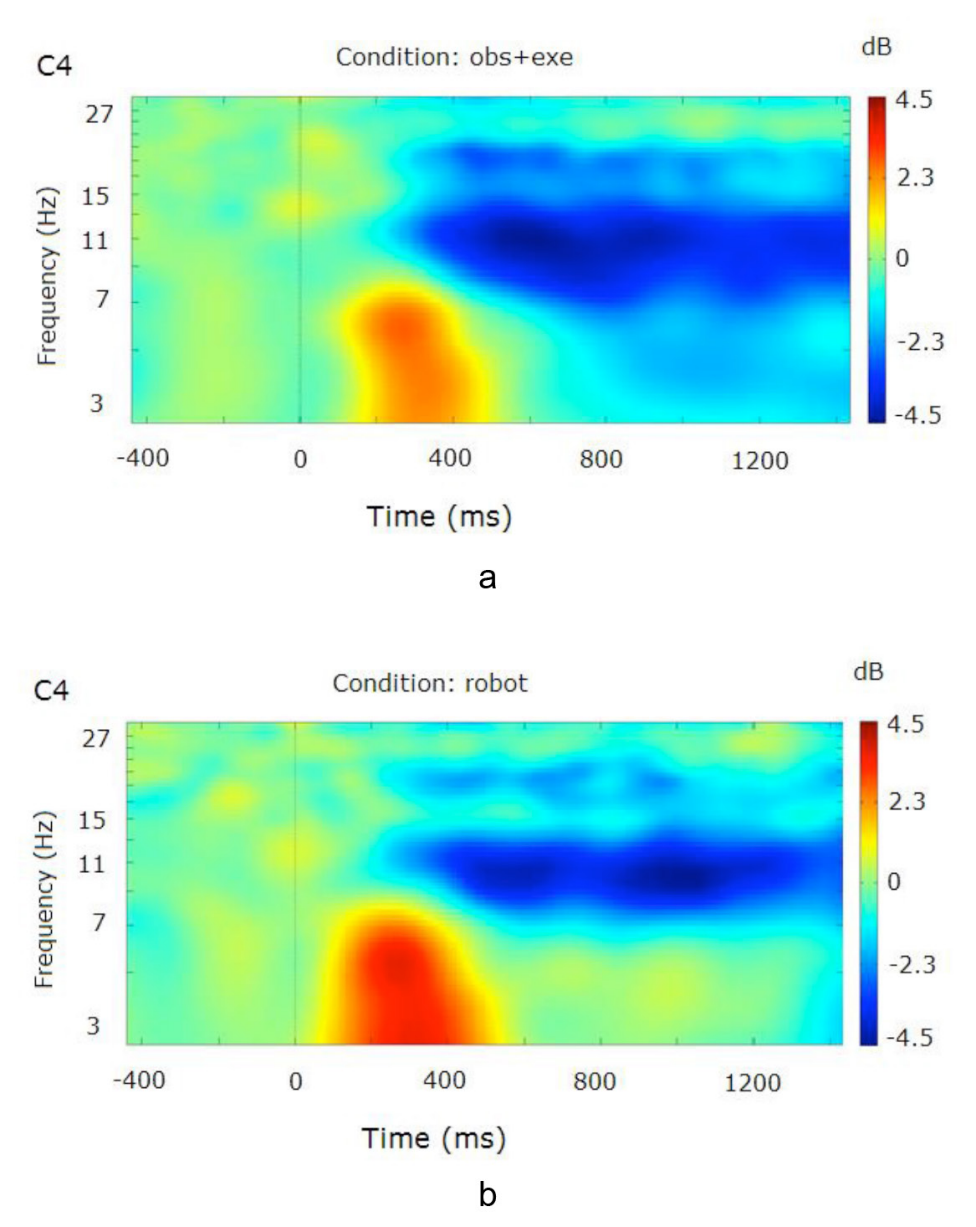
Time-frequency plots for two conditions (obs+exe (a) and robot (b)) at channel C4 (right hemisphere). Plots for the left hemisphere (C3) were similar and are not shown. The frequency axis is log scaled. The zero point on the time axis indicates the onset of the video stimuli.

Over the somatosensory cortex, time-frequency analysis of the relevant channels led to a decrease in mu (8**–**12 Hz) and beta (15**–**25 Hz) band power shortly after stimulus onset starting around 300 ms (Figure 7).****

In mu band, a 2 (executional condition: obs+exe vs. robot) by 2 (hemisphere: left vs. right) within subjects analysis of variance over central channels (C3, C4) revealed no significant main effects of executional condition [F(1,7) = 0.377, P > 0.5] nor hemisphere [F(1,7) = 0.254, P > 0.5], nor a significant presence of executional condition by hemisphere interaction [F(1,7) = 0.453, P > 0.5]. Similar 2 (executional condition) by 2 (hemisphere) repeated measures ANOVAs at frontal (F7,F8) and parietal channels (P3,P4) revealed no main effects or interactions {F7–F8: executional condition [F(1,7) = 1.918], hemisphere [F(1,7) = 0.052], executional condition x hemisphere [F(1,7) = 0.647]; P3–P4: executional condition [F(1,7) = 1.887], hemisphere [F(1,7) = 1.176], executional condition x hemisphere [F(1,7) = 1.449], all P > 0.1}.

For beta frequency band, a similar 2 (executional condition: obs vs. no robot) by 2 (hemisphere: left vs. right) repeated measures ANOVAs were utilized. There were no main effects or interactions {C3–C4: executional condition [F(1,7) = 3.532], hemisphere [F(1,7) = 0.003], executional condition x hemisphere [F(1,7) = 3.836]; F7–F8: executional condition [F(1,7) = 0.637], hemisphere [F(1,7) = 0.276], executional condition x hemisphere [F(1,7) = 0.664]; P3–P4: executional condition [F(1,7) = 3.695], hemisphere [F(1,7) = 0.316], executional condition x hemisphere [F(1,7) = 0.176], all P > 0.1}.

### 3.4. Effect of observation of hand movement stimuli with different kinetics

**Figure 8:**
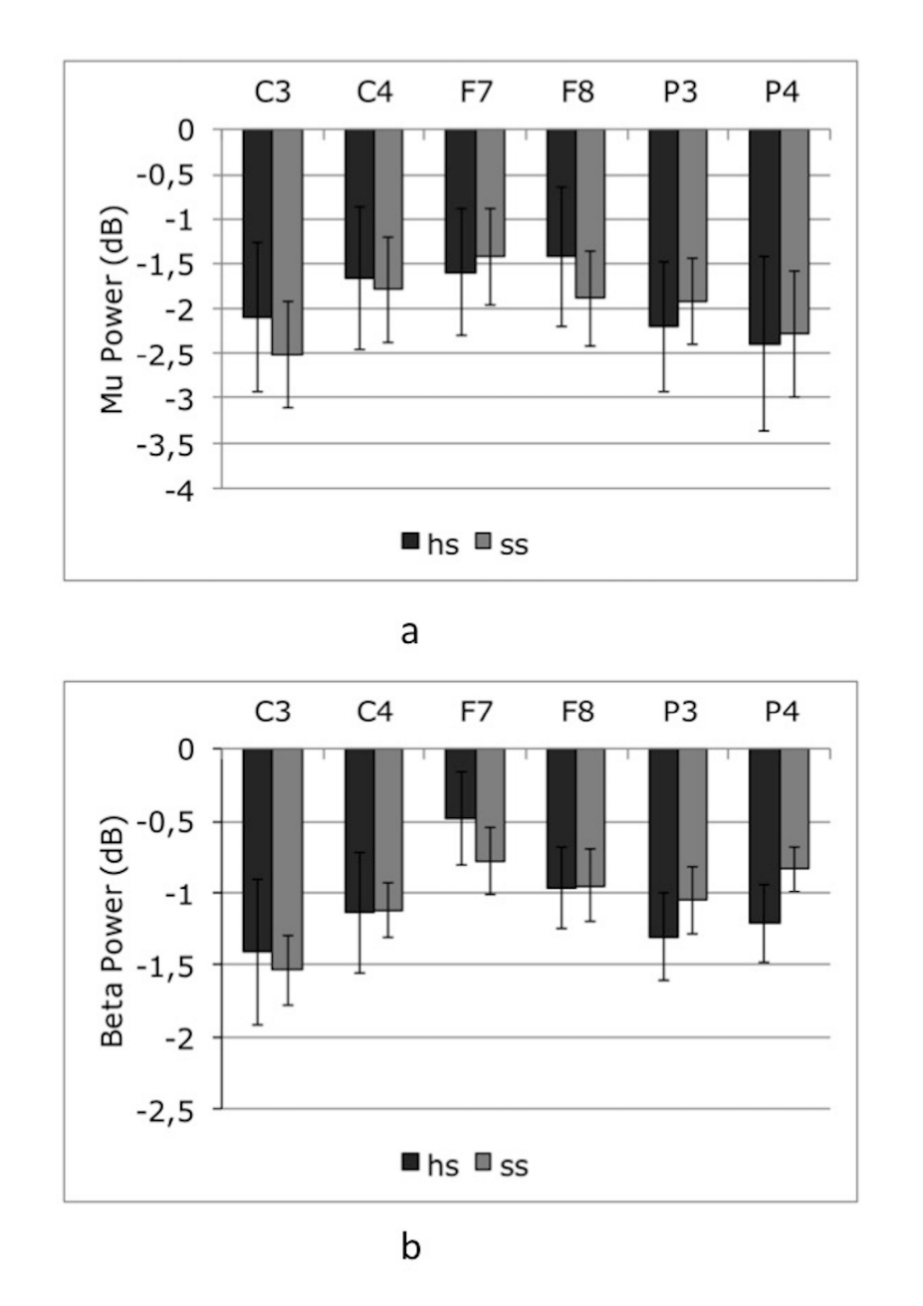
Mu (a) and beta (b) band suppression to experimental conditions (hs, ss). Bars represent the mean log ratio of power in the mu (8-12 Hz) and beta (15-25 Hz) frequency bands during session 1 (hs:hard spring, ss: soft spring). Error bars indicate the standard error of the mean. A log ratio less than zero indicates mu/beta suppression.

For mu and beta frequencies a 2 way ANOVA [spring type (hard, soft by hemisphere (right, left)] was performed at frontal (F7, F8) channels (Figure 8). 

Mu suppression

Mu oscillations did not reveal a main effect of spring type [F (1,7) = 0.145, P > 0.1] or hemisphere [F(1,7) = 0.178, P > 0.1] but the spring type x hemisphere interaction was at the cusp of significance [F(1,7) = 5.303, P = 0.054].

Beta suppression

Beta oscillations did not reveal significant main effects or interactions {spring type: [F(1,7) = 1.012], hemisphere [F(1,7) = 2.841], hemisphere X spring type interaction [F(1,7) = 1.047] all P > 0.1}.

## 4. Discussion

This study investigated how the EEG Mu/Beta rhythm that is considered to index human MNS activity is modulated by an anticipatory effect of execution. The study model was based on two critical assumptions: the mere observation condition (obs) in session 1 was matched with the no robot condition in session 3, and obs+exe condition in session 2 was matched with robot condition in session 3. This approach in the experimental design reveals the possible anticipatory effect of robot accompany on observation and execution. 

In the current study only the obs+exe and robot conditions include execution of the left hand. This execution was initiated and terminated by ExoPinch finger robot and therefore the action is not a voluntary self-initiated motion of the individual. It was previously suggested that the MNS is not selective for volitional hand actions [47]. Additionally, passive movements share much of the motor control circuitry used for voluntary movements [20]. It is then plausible to think that similar neural circuits might be active during the execution of the finger by the exoskeleton robot.

The principal finding in this study was that an anticipatory effect of execution might modulate the existing neural activity during action observation over the sensorimotor areas, which might further suggest the preparatory processes of cortical motor areas.

### 4.1. Mu/Beta suppression in cortical motor areas

In the obs condition subjects knew that they would merely observe the stimuli without any robot accompany (it was absent in the experiment area) and similarly in the obs+exe condition, the finger robot ExoPinch would certainly and simultaneously present in each experimental trial. None of the conditions presented unexpectedly to the subjects in these first two sessions. 

All the experimental conditions (obs, obs+exe, robot, no robot) resulted in significant attenuations in the power of mu and beta band oscillations over somatosensory cortex indicating more desynchronized neural assemblies. Consistent with several previous work [3,36], larger suppressions of mu/beta band power have been found over the somatosensory cortex (C3, C4) than other electrode locations for all conditions of the experiment. Additionally, obs+exe condition had a larger magnitude of MNS activity over all relevant electrode sides than obs condition. In other words, mu and beta band suppressions were larger when execution and observation were performed simultaneously. This finding supports several previous studies that execution has a relatively stronger effect on mu/beta suppression than observation [35]. However, for robot and no robot conditions such evidence of a difference in magnitude was evident only over sensorimotor cortex. This result indicates conditional differences (execution, observation) of mu/beta suppression over central area of the cortex [48]. In this topic one may argue that EEG mu suppression may not reflect MSN activity. Although this topic is still debated, recently it has been shown in a metaanalysis that EEG suppression is a powerful indicator of mirror neuron activity [49].

### 4.2. Effect of anticipation on observation and execution

In this experiment, an interaction effect was found between the observational condition (obs, no robot) and the hemisphere (C3, C4) at mu frequency band. In these two conditions, subjects were merely observing the video stimuli without any overt movement. It is likely that an anticipation of the robot accompany modulated the neural activity at mu frequency band over somatosensory areas while subjects were merely watching the video clips.

The exact synchronization of the observation and execution session (obs+exe) was preceded by the random accompany session. Therefore, subjects knew the exact motor movement (flexion/extension of the index finger) in this session while matching or “mirroring” a motor response to visual stimulus. The same neural mirroring mechanism might be active during the last session of the experiment. The behavioral changes related to anticipation in this session correlates with changes in functional activation in cortical motor areas [50]. This anticipatory effect might be preparing the central brain regions in planning of a visually guided finger movement by engaging the mirror neuron mechanism [51]. 

Although our main hypotheses primarily related to the anticipatory effect of execution on observation, we also analyzed obs+exe and robot conditions over the channels of interest to explore any modulatory effect of anticipation on execution. We did not find any main or interaction effects in these analyses. Since the anticipated action (flexion/extension of their left hand’s index finger) is already known by the subjects due to our experimental design, the anticipatory effect of execution on its counterpart might not have a remarkable effect. 

### 4.3. Effect of visual stimuli on MNS

In the current study, analysis of visual stimuli with different kinetical properties (hs, ss) revealed that the attenuation of mu suppression was significantly modulated by the visual stimuli ss and hs at frontal channels. It is likely that the neural activity might be context-dependent in the corresponding brain area, ventral premotor cortex (vPMC) [45], and that changes in attenuation may occur depending on the required force in the task. Indeed, vMPC is strongly connected to M1 [12] and action upon the object is necessary to trigger the encoding of force requirements in the motor system [42]. Overall, modulation of mu suppression at frontal channels is likely to be associated with the observed force requirement. 

The kinematical features of the objects were not considered as an independent factor since the springs were fabricated at exactly the same length and therefore the grasp dimensions (the grasp width of the objects hs and ss) were identical in the video clips. 

### 4.4. MNS and rehabilitation 

It is now a well-accepted notion that mere observation of actions activates the corresponding motor representations of those actions in the brain [52]. The neurorehabilitative use of this approach is based on the discovery of the putative mirror neuron system and their functional abilities.

The main mechanism of the activation of the MNS is supposed to be an internal simulation of the observed action. Using an internal simulation of an action, an individual can reactivate the action representations previously stored in the brain [15, 30]. 

Our approach in the experimental design serves as a novel framework for an action-observation based treatment of stroke. The observation of action with a concomitant effect of anticipation may initiate the related motor pathways of the observed action by mostly simulating the corresponding motor act. Furthermore, the type of the observed stimuli (e.g., hand-object interaction, kinematical or kinetical parameters) may have a relatively strong effect on MNS activity. In this study, investigation of the effect of the visual stimuli on MNS revealed the main effect of video stimuli of hand squeezing soft and hard springs, at the frontal channels corresponding nearly to ventral premotor cortex area of the brain. The activation of mirror neurons in premotor cortex during action observation plays a crucial role in observational learning [11].

The combination of motor exercise and action observation seems to constitute a powerful approach for neurorehabilitation of motor deficits following stroke [53]. An anticipatory effect of execution may well facilitate the observation session and this would allow a better eventual action execution performance. The common neural network for action observation and execution (mirror neuron system), in this sense, increases the value of the treatment of poststroke motor disabilities.

Based on our results, we propose that specific type of visual stimuli can be combined with the functional abilities of the MNS in the action observation based treatment of hand motor dysfunction of stroke patients to have a positive additional impact. 

## Acknowledgements

This work was supported by the Scientific and Technological Research Council of Turkey (TÜBİTAK) under Grant 114E621.
